# 1-Aryl-3-[4-(thieno[3,2-*d*]pyrimidin-4-yloxy)phenyl]ureas as VEGFR-2 Tyrosine Kinase Inhibitors: Synthesis, Biological Evaluation, and Molecular Modelling Studies

**DOI:** 10.1155/2013/154856

**Published:** 2013-07-07

**Authors:** Pedro Soares, Raquel Costa, Hugo J. C. Froufe, Ricardo C. Calhelha, Daniela Peixoto, Isabel C. F. R. Ferreira, Rui M. V. Abreu, Raquel Soares, Maria-João R. P. Queiroz

**Affiliations:** ^1^Centro de Química, Escola de Ciências, Universidade do Minho, Campus de Gualtar, 4710-057 Braga, Portugal; ^2^CIQ/Departamento de Química e Bioquímica, Faculdade de Ciências, Universidade do Porto, Rua do Campo Alegre, 4169-007 Porto, Portugal; ^3^Departamento de Bioquímica (U38-FCT), Faculdade de Medicina, Universidade do Porto, 4200-319 Porto, Portugal; ^4^Centro de Investigação de Montanha (CIMO), Escola Superior Agrária, Instituto Politécnico de Bragança, Campus de Santa Apolónia, Apartado 1172, 5301-855 Bragança, Portugal

## Abstract

The vascular endothelial growth factor receptor-2 (VEGFR-2) is a tyrosine kinase receptor involved in the growth and differentiation of endothelial cells that are implicated in tumor-associated angiogenesis. In this study, novel 1-aryl-3-[4-(thieno[3,2-*d*]pyrimidin-4-yloxy)phenyl]ureas were synthesized and evaluated for the VEGFR-2 tyrosine kinase inhibition. Three of these compounds showed good VEGFR-2 inhibition presenting low IC_50_ values (150–199 nM) in enzymatic assays, showing also a significant proliferation inhibition of VEGF-stimulated human umbilical vein endothelial cells (HUVECs) at low concentrations (0.5–1 *µ*M), using the Bromodeoxyuridine (BrdU) assay, not affecting cell viability. The determination of the total and phosphorylated (active) VEGFR-2 was performed by western blot, and it was possible to conclude that the compounds significantly inhibit the phosphorylation of the receptor at 1 *µ*M pointing to their antiproliferative mechanism of action in HUVECs. The molecular rationale for inhibiting the tyrosine kinase domain of VEGFR-2 was also performed and discussed using molecular docking studies.

## 1. Introduction

**Figure 1:**
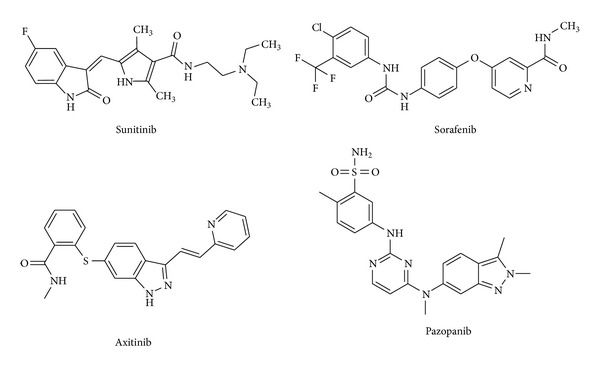
Competitive inhibitors of VEGFR-2 in clinical use against several cancers: sunitinib (Sutent, C. P. Pharmaceuticals International, NY, USA), soranefib (Nexavar, Bayer, Germany), axitinib (Inlyta, Pfizer), and pazopanib (Votrient, GlaxoSmithKline).

Angiogenesis is the process of new blood vessel formation from preexisting vascular networks by capillary sprouting [1] and plays an important role in the pathogenesis of several disorders including cancer, vasculoproliferative ocular disorders, and rheumatoid arthritis [2]. A key regulatory pathway of angiogenesis is mediated by the vascular endothelial growth factor (VEGF), involved in the vascular permeability and an inducer of endothelial cell proliferation, migration, and survival [3], and its cell membrane tyrosine kinase receptor VEGFR-2 (also known as KDR) [4]. Upon ligand binding, VEGFR-2 undergoes autophosphorylation, triggering signaling pathways leading to endothelial cell proliferation and subsequent angiogenesis. Small molecule inhibitors act by competing with ATP for its binding site of the VEGFR-2 intracellular tyrosine kinase domain, thereby preventing the signaling pathways that lead to angiogenesis [5]. Several small molecule VEGFR-2 inhibitors have emerged as promising antiangiogenic agents for possible treatment against a wide variety of cancers (Figure 1). Sunitinib was approved for the treatment of renal cell carcinoma and gastrointestinal stromal tumor, and Sorafenib was approved for the treatment of primary kidney cancer and hepatocellular carcinoma [6]. Recently, two new VEGFR-2 inhibitors have been approved for the treatment of advanced renal cell carcinoma: axitinib [7] and pazopanib [8]. A number of thienopyridines and thienopyrimidines ureas have also shown potent VEGFR-2 inhibition activity [9, 10] including arylether derivatives [11–13].

In this report, we describe an ongoing effort to develop novel small molecules as VEGFR-2 inhibitors, based on the aryletherthieno[3,2-*d*]pyrimidine arylurea scaffold. The synthesis of the compounds and the VEGFR-2 tyrosine kinase phosphorylation inhibition evaluation using either enzymatic or cellular assays including the determination of the total and of the phosphorylated VEGFR-2 by western blot were performed. The probable binding mode of the 1-aryl-3-[4-(thieno[3,2-*d*]pyrimidin-4-yloxy)phenyl]ureas with the receptor using docking studies is also presented and discussed.

## 2. Materials and Methods

### 2.1. Synthesis

Melting points (°C) were determined in a Stuart SMP3 and are uncorrected.  ^1^H and  ^13^C NMR spectra were recorded on a Varian Unity Plus at 300 and 75.4 MHz, respectively, or on a Bruker Avance III at 400 and 100.6 MHz, respectively. Two-dimensional ^1^H-^13^C correlations were performed to attribute some signals. Mass spectra (MS) EI-TOF or ESI-TOF and HRMS on the M^+^, [M + H]^+^ or on [M + Na]^+^, were performed by the mass spectrometry service of the University of Vigo, C.A.C.T.I., Spain. 

The reactions were monitored by thin layer chromatography (TLC) using Macherey-Nagel precoated aluminium silica gel 60 sheets (0.20 mm) with UV_254_ indicator. Column chromatography was performed on Panreac, silica gel 60, 230–400 mesh. 

#### 2.1.1. General Procedure for the Synthesis of Compounds 1a and 1b

In a flask with 5 mL of DMF, thienopyrimidine (1 equiv.), 4-aminophenol (1 equiv.), and K_2_CO_3_ (4 equiv.) were heated at 140°C for 2 h. After cooling, water (5 mL) and ethyl acetate (5 mL) were added. The phases were separated, and the aqueous phase was extracted with more ethyl acetate (2 × 5 mL). The organic phase was dried (MgSO_4_) and filtered. The solvent was evaporated under reduced pressure giving a solid which was submitted to column chromatography.

*4-(Thieno[3,2-d]pyrimidin-4-yloxy)aniline *(***1a***). 4-Chlorothieno[3,2-*d*]pyrimidine (242 mg, 1.42 mmol) and 4-aminophenol (155 mg, 1.42 mmol) were heated, and the reaction mixture was treated according to the general procedure. Column chromatography using ethyl acetate gave compound **1a** as a yellow solid (290 mg, 91%), m.p. 115.8–116.5°C.  ^1^H NMR (300 MHz, DMSO-*d*_6_): *δ* 5.15 (s, 2H, NH_2_), 6.60 (d, *J* = 9.0 Hz, 2H, 2 and 6-H), 6.95 (d, *J* = 9.0 Hz, 2H, 3 and 5-H), 7.62 (d, *J* = 5.6 Hz, 1H, HetAr), 8.40 (d, *J* = 5.6 Hz, 1H, HetAr), 8.66 (s, 1H, 2′-H) ppm.  ^13^C NMR (75.4 MHz, DMSO-*d*_6_): *δ* 114.2 (2 and 6-CH), 116.6 (C), 122.3 (3 and 5-CH), 124.1 (CH), 137.1 (CH), 141.7 (C), 146.9 (C), 154.2 (2′-CH), 162.9 (C), 164.3 (C) ppm. MS (EI-TOF) *m*/*z* (%): 243.05 (M^+^, 100) HRMS (EI-TOF): calcd for C_12_H_9_N_3_OS [M^+^] 243.0466, found 243.0467.

*4-(7-Methylthieno[3,2-d]pyrimidin-4-yloxy)aniline *(***1b***). 4-Chloro-7-methylthieno[3,2-*d*]pyrimidine (242 mg, 1.31 mmol) and 4-aminophenol (143 mg, 1.31 mmol) were heated, and the reaction mixture was treated according to the general procedure. Column chromatography using ethyl acetate gave compound **1b** as a yellow solid (310 mg, 92%), m.p. 167.8–168.6°C.  ^1^H NMR (300 MHz, DMSO-*d*_6_): *δ* 2.40 (s, 3H, CH_3_), 5.13 (s, 2H, NH_2_), 6.61 (d, *J* = 9.0 Hz, 2H, 2 and 6-H), 6.93 (d, *J* = 9.0 Hz, 2H, 3 and 5-H), 8.01 (s, 1H, 6′-H), 8.67 (s, 1H, 2′-H) ppm.  ^13^C NMR (75.4 MHz, DMSO-*d*_6_): *δ* 12.5 (CH_3_), 114.2 (2 and 6-CH), 116.8 (C), 122.3 (3 and 5-CH), 131.4 (6′-CH), 132.6 (C), 141.7 (C), 146.8 (C), 153.9 (2′-CH), 161.8 (C), 164.3 (C) ppm. MS (EI-TOF) *m*/*z* (%): 257.06 (M^+^, 100) HRMS (EI-TOF): calcd for C_13_H_11_N_3_OS [M^+^] 257.0623, found 257.0621.

#### 2.1.2. General Procedure for the Synthesis of 1,3-Diarylureas 2a–f

Compounds **1a** or **1b** and different arylisocyanates (1 equiv.) in 6 mL CH_2_Cl_2_ : THF (1 : 1) were left stirring at room temperature for 16 h. If a precipitate does not come out after this time, hexane (3–5 mL) is added to the mixture to precipitate the product. This was filtered under vacuum to give the corresponding 1,3-diarylureas.

*1-Phenyl-3-[4-(thieno[3,2-d]pyrimidin-4-yloxy)phenyl]urea *(***2a***). From compound **1a** (100 mg, 0.410 mmol), and phenylisocyanate (0.0400 mL, 0.410 mmol) compound **2a** was isolated as a white solid (117 mg, 79%), m.p. 237.3–238.7°C.  ^1^H NMR (400 MHz, DMSO-*d*_6_): *δ* 6.95–6.99 (m, 1H, Ar-H), 7.24–7.30 (m, 4H, Ar-H), 7.45–7.47 (m, 2H, 2 × Ar-H), 7.54 (d, *J* = 9.0 Hz, 2H, 2 × Ar-H), 7.65 (d, *J* = 5.2 Hz, 1H, HetAr), 8.45 (d, *J* = 5.2 Hz, 1H, HetAr), 8.68 (s, 1H, NH), 8.69 (s, 1H, 2′′′-H), 8.78 (s, 1H, NH) ppm.  ^13^C NMR (100.6 MHz, DMSO-*d*_6_): *δ* 116.8 (C), 118.2 (2 × CH), 119.2 (2 × CH), 121.8 (CH), 122.3 (2 × CH), 124.2 (CH), 128.8 (2 × CH), 137.2 (CH), 137.6 (C), 139.6 (C), 146.0 (C), 152.6 (C), 154.1 (2′′′-CH), 163.0 (C), 163.8 (C) ppm. MS (ESI-TOF) *m*/*z* (%): 363.09 ([M + H]^+^, 100) HRMS (ESI-TOF): calcd for C_19_H_15_N_4_O_2_S [M + H]^+^ 363.0910, found 363.0909.

*1-(4-Methoxyphenyl)-3-[4-(thieno[3,2-d]pyrimidin-4-yloxy)phenyl]urea *(***2b***). From compound **1a** (100 mg, 0.410 mmol), and 4-methoxyphenylisocyanate (0.0500 mL, 0.410 mmol) compound **2b** was isolated as a white solid (145 mg, 90%), m.p. 247.2–248.3°C. ^1^H NMR (400 MHz, DMSO-*d*_6_): *δ* 3.71 (s, 3H, OCH_3_), 6.86 (d, *J* = 9.0 Hz, 2H, 3′ and 5′-H), 7.23 (d, *J* = 9.0 Hz, 2H, 2 × Ar-H), 7.36 (d, *J* = 9.0 Hz, 2H, 2′ and 6′-H), 7.53 (d, *J* = 9.0 Hz, 2H, 2 × Ar-H), 7.65 (d, *J* = 5.6 Hz, 1H, HetAr), 8.44 (d, *J* = 5.6 Hz, 1H, HetAr), 8.50 (s, 1H, NH), 8.69 (s, 1H, 2′′′-H), 8.70 (s, 1H, NH) ppm.  ^13^C NMR (100.6 MHz, DMSO-*d*_6_): *δ* 55.1 (OCH_3_), 114.0 (3′ and 5′-CH), 116.8 (C), 119.1 (2 × CH), 120.1 (2′ and 6′-CH), 122.3 (2 × CH), 124.2 (CH), 132.7 (C), 137.2 (CH), 137.8 (C), 145.9 (C), 152.8 (C), 154.1 (2′′′-CH), 154.5 (C), 163.0 (C), 163.8 (C) ppm. MS (ESI-TOF) *m*/*z* (%): 393.08 ([M+H]^+^, 39) HRMS (ESI-TOF): calcd for C_20_H_17_N_4_O_3_S [M+H]^+^ 393.1016, found 393.1026.

*1-(4-Cyanophenyl)-3-[4-(thieno[3,2-d]pyrimidin-4-yloxy)phenyl]urea *(***2c***). From compound **1a** (100 mg, 0.410 mmol) and 4-cyanophenylisocyanate (59.0 mg, 0.410 mmol), compound **2c** was isolated as a white solid (111 mg, 70%), m.p. 245.5–247.2°C.  ^1^H RMN (300 MHz, DMSO-*d*_6_): *δ* 7.26 (d, *J* = 9.2 Hz, 2H, 2 × Ar-H), 7.55 (d, *J* = 9.2 Hz, 2H, 2 × Ar-H), 7.62–7.66 (m, 3H, 2′ and 6′-H and HetAr), 7.73 (d, *J* = 9.2 Hz, 2H, 3′ and 5′-H), 8.45 (d, *J* = 5.2 Hz, 1H, HetAr), 8.69 (s, 1H, 2′′′-H), 8.99 (s, 1H, NH), 9.24 (s, 1H, NH) ppm.  ^13^C NMR (75.4 MHz, DMSO-*d*_6_): *δ* 103.3 (C), 116.8 (C), 118.0 (2′ and 6′-CH), 119.3 (C), 119.7 (2 × CH), 122.4 (2 × CH), 124.3 (CH), 133.3 (3′ and 5′-CH), 137.0 (C), 137.2 (CH), 144.2 (C), 146.5 (C), 152.2 (C), 154.1 (2′′′-CH), 163.0 (C), 163.8 (C) ppm. MS (ESI-TOF) *m*/*z* (%): 388.09 ([M + H]^+^, 100) HRMS (ESI-TOF): calcd for C_20_H_13_N_5_O_2_S [M + H]^+^ 388.0863, found 388.0861.

*1-Phenyl-3-[4-(7-Methylthieno[3,2-d]pyrimidin-4-yloxy)phenyl]urea *(***2d***). From compound **1b** (100 mg, 0.389 mmol) and phenylisocyanate (0.0400 mL, 0.389 mmol), compound **2d** was isolated as a white solid (121 mg, 83%), m.p. 285.7–287.3°C.  ^1^H NMR (400 MHz, DMSO-*d*_6_): *δ* 2.43 (s, 3H, CH_3_), 6.96 (m, 1H, Ar-H), 7.23–7.29 (m, 4H, Ar-H), 7.46 (m, 2H, 2 × Ar-H), 7.53 (d, *J* = 9.2 Hz, 2H, 2 × Ar-H), 8.10 (s, 1H, 6′′′-H), 8.70 (s, 1H, NH), 8.71 (s, 1H, 2′′′-H), 8.78 (s, 1H, NH) ppm.  ^13^C NMR (100.6 MHz, DMSO-*d*_6_): *δ* 12.5 (CH_3_), 116.9 (C), 118.2 (2 × CH), 119.2 (2 × CH), 121.8 (C), 122.3 (2 × CH), 128.8 (2 × CH), 131.5 (6′′′-CH), 132.7 (C), 137.6 (C), 139.6 (C), 146.1 (C), 152.6 (C), 153.9 (2′′′-CH), 161.9 (C), 163.9 (C) ppm. MS (ESI-TOF) *m*/*z* (%): 377.10 ([M + H]^+^, 100) HRMS (ESI-TOF): calcd for C_20_H_17_N_4_O_2_S [M + H]^+^ 377.1067, found 377.1064.

*1-(4-Methoxyphenyl)-3-[4-(7-methylthieno[3,2-d]pyrimidin-4-yloxy)phenyl]urea *(***2e***). From compound **1b** (100 mg, 0.389 mmol) and 4-methoxyphenylisocyanate (0.0500 mL, 0.389 mmol), compound **2e** was isolated as a white solid (131 mg, 83%), m.p. 248.4–249.6°C.  ^1^H NMR (400 MHz, DMSO-*d*_6_): *δ* 2.43 (s, 3H, CH_3_), 3.71 (s, 3H, OCH_3_), 6.86 (d, *J* = 9.2 Hz, 2H, 3′ and 5′-H), 7.22 (d, *J* = 9.2 Hz, 2H, 3′′ and 5′′-H), 7.36 (d, *J* = 9.2 Hz, 2H, 2′ and 6′-H), 7.52 (d, *J* = 9.2 Hz, 2H, 2′′ and 6′′-H), 8.06 (s, 1H, 6′′′-H), 8.49 (s, 1H, NH), 8.69 (s, 1H, NH), 8.70 (s, 1H, 2′′′-H) ppm.  ^13^C NMR (100.6 MHz, DMSO-*d*_6_): *δ* 12.5 (CH_3_), 55.2 (OCH_3_), 114.0 (3′ and 5′-CH), 116.9 (C), 119.1 (2′′ and 6′′-CH), 120.1 (2′ and 6′-CH), 122.2 (3′′ and 5′′-CH), 131.5 (6′′′-CH), 132.7 (C), 137.8 (C), 146.0 (C), 152.8 (C), 153.9 (2′′′-CH), 154.5 (C), 161.9 (C), 163.9 (C) ppm. MS (ESI-TOF) *m*/*z* (%): 407.12 ([M + H]^+^, 100) HRMS (ESI-TOF): calcd for C_21_H_19_N_4_O_3_S [M + H]^+^ 407.1172, found 407.1182.

*1-(4-Cyanophenyl)-3-[4-(7-methylthieno[3,2-d]pyrimidin-4-yloxy)phenyl]urea *(***2f***). From compound **1b** (100 mg, 0.389 mmol) and 4-cyanophenylisocyanate (56.0 mg, 0.389 mmol), compound **2f** was isolated as a white solid (128 mg, 82%), m.p. 246.7–248.2°C.  ^1^H NMR (400 MHz, DMSO-*d*_6_): *δ* 2.42 (s, 3H, CH_3_), 7.25 (d, *J* = 9.2 Hz, 2H, 3′′ and 5′′-H), 7.54 (d, *J* = 9.2 Hz, 2H, 2′′ and 6′′-H), 7.64 (d, *J* = 9.2 Hz, 2H, 2′ and 6′-H), 7.72 (d, *J* = 9.2 Hz, 2H, 3′ and 5′-H), 8.06 (s, 1H, 6′′′-H), 8.70 (s, 1H, 2′′′-H), 8.97 (s, 1H, NH), 9.23 (s, 1H, NH) ppm.  ^13^C NMR (100.6 MHz, DMSO-*d*_6_): *δ* 12.5 (CH_3_), 103.3 (C), 117.0 (C), 118.1 (2′ and 6′-CH), 119.3 (C), 119.7 (2′′ and 6′′-CH), 122.4 (3′′ and 5′′-CH), 131.5 (6′′′-CH), 132.7 (C), 133.3 (3′ and 5′-CH), 137.0 (C), 144.2 (C), 146.6 (C), 152.2 (C), 153.9 (2′′′-CH), 162.0 (C), 163.8 (C) ppm. MS (ESI-TOF) *m*/*z* (%): 402.10 ([M + H]^+^, 100) HRMS (ESI-TOF): calcd for C_21_H_16_N_5_O_2_S [M + H]^+^ 402.1019, found 402.1029.

### 2.2. VEGFR-2 Enzymatic Inhibition Assay

The compounds were assessed for VEGFR-2 inhibition activity using the *Z*′-LYTE-Tyr1 Peptide assay kit (according to the procedures recommended by the manufacturer Invitrogen, Cat. PV3190) [14]. Briefly, assays were performed in a total of 20 *μ*L in 384-well plates using fluorescence resonance energy transfer technology. A Tyr1 substrate (coumarin-fluorescein double-labeled peptide) at 1.0 *μ*M was incubated for 1 h with 4 *μ*g/mL VEGFR-2, 10 *μ*M ATP, and inhibitors at room temperature in 50 mM Hepes/Na (pH 7.5), 10 mM MgCl_2_, 2.0 mM MnCl_2_, 2.5 mM DTT, 0.10 mM orthovanadate, and 0.01% bovine serum albumin. The wells were incubated at 25°C for 1 hour and 5.0 *μ*L of the development reagent was added to each well. After a second incubation of 1 hour, a stop reagent was added to each well. Using a Biotek FLX800 microplate, the fluorescence was read at 445 nm and 520 nm (excitation 400 nm), and Gen5 Software was used for data analysis. The validation assay was performed using Staurosporine that presented a IC_50_ value 6 nM that compares to the one reported in the literature (IC_50_ 7 nM) [14].

### 2.3. Cell Culture Experiments Using HUVECs

#### 2.3.1. Cell Cultures

Human umbilical vein endothelial cells (HUVECs) were obtained from the ScienceCell Research Labs (San Diego, CA, USA), HUVECs were seeded on plates coated with 0.2% gelatin (Sigma-Aldrich, Sintra, Portugal) and cultured in M199 medium (Sigma-Aldrich, Sintra, Portugal) supplemented with 20% fetal bovine serum (FBS) (Invitrogen Life Technologies, Scotland, UK), 1% penicillin/streptomycin (Invitrogen Life Technologies), 0.01% heparin (Sigma-Aldrich), and 30 mg/mL endothelial cell growth supplement (Biomedical Technologies Inc., MA, USA) and maintained at 37°C in a humidified 5% carbon dioxide atmosphere. Cells were kept between passages 3 and 8 for every experiment. The test compounds were dissolved in DMSO and added to cell cultures at a concentration of 0.1–10 *μ*M. Incubations were performed for 24 h in medium supplemented with 2% FBS, 1% penicillin/streptomycin, and 60 ng/mL of the vascular endothelial growth factor (Sigma-Aldrich, Sintra, Portugal). Control cells were incubated with vehicle (DMSO at 0.1% in every culture).

#### 2.3.2. MTS Toxicity Assay

HUVECs (2 × 10^5^ cells/mL) were allowed to grow for 24 h and then incubated with the test compounds at a range concentration between 0.1 and 10 *μ*M or control (0.1% DMSO) for 24 h. After the incubation period, cells were washed and their viability was assessed using Cell Titer 96 Aqueous ONE Solution Reagent MTS [3-(4,5-dimethylthiazol-2-yl)-5-(3-carboxymethoxyphenyl)-2-(4-sulfophenyl)-2H-tetrazolium] colorimetric assay (Promega, Madison, USA), according to the instructions provided by the manufacturer and as previously described [15]. Optical density was measured at 492 nm. Three independent experiments were performed, and the results were expressed as mean ± SEM.

#### 2.3.3. BrdU Incorporation Assay

HUVECs (6 × 10^4^ cells/mL) were grown during 24 h and then were incubated with the compounds at 0.1–10 *μ*M or control (0.1% DMSO) for 24 h. Cells were also incubated with 5′-bromodeoxyuridine (BrdU), a thymidine analogue which incorporates into DNA of dividing cells. After incubation with BrdU solution at a final concentration of 0.01 mM during the treatment period, the optical density of proliferating cells (positive for BrdU) after ELISA assay using anti-BrdU-specific antibodies (Roche Diagnostics, Mannheim, Germany) was evaluated at the microplate reader according to the manufacturer's instructions and as previously reported [16]. The results are given as percentage versus control group (100%).

#### 2.3.4. Cell Apoptosis

HUVECs (6 × 10^4^ cells/mL) were grown for 24 h on glass coverslips and incubated with different concentrations (0.1–1 *μ*M) of the tested compounds. TUNEL assay was performed using the In Situ Cell Death Detection kit (Roche Diagnostics, Mannheim, Germany), according to the manufacturer's instruction and as previously reported [16]. Immunofluorescence was visualized under a fluorescence microscope (Olympus, BH-2, UK). The percentage of stained cells was evaluated by counting the cells stained with TUNEL (apoptotic cells) divided by the total number of nuclei counterstained with DAPI (Invitrogen, CA, USA) at a 200x magnification field. One thousand nuclei were evaluated. Results were expressed as percentage of control (100%).

#### 2.3.5. Western Blotting Analyses

Proteins were isolated from HUVEC lysates using RIPA (Chemicon International, CA, USA). Proteins were quantified using a spectrophotometer (Jenway, 6405 UV/vis, Essex, UK), and 20 *μ*g of protein were subjected to 8% SDS-PAGE with a 5% stacking gel. After electrophoresis, proteins were blotted into a Hybond nitrocellulose membrane (Amersham, Arlington, VA, USA). Immunodetection for total VEGFR-2 (1 : 1000; Cell Signaling, MA, USA), phosphorylated (activated) VEGFR-2 (1 : 750; Santa Cruz Biotechnology, CA, USA), and *β*-actin (1 : 3000; Abcam, Cambridge, UK) was accomplished with enhanced chemiluminescence (ECL kit, Amersham, Arlington, USA). The phosphorylated antibody recognizes Tyr951 kinase insert domain, a major site of VEGFR-2 phosphorylation particularly involved in angiogenic processes. The relative intensity of each protein blotting analysis was measured using a computerized software program (Bio-Rad, CA, USA), and the expression of activated and total VEGFR-2 were normalized with total VEGFR-2 and *β*-actin bands, respectively, to compare the expression of proteins in different treatment groups. 

#### 2.3.6. Statistical Analyses

All experiments were performed at least in three independent experiments. Quantifications are expressed as mean ± SEM. Statistical significance of difference between the distinct groups was evaluated by analysis of variance (ANOVA) followed by the Bonferroni test. A difference between experimental groups was considered significant with a confidence interval of 95%, whenever *P* < 0.05.

### 2.4. Docking Simulations Using AutoDock4

A VEGFR-2 crystal structure (PDB: 2XIR) was extracted from the Protein Data Bank (PDB) (http://www.rcsb.org/). The cocrystallized ligand was extracted from the PDB file, and AutoDockTools was used to assign polar hydrogens and Gasteiger charges to the compounds [17]. AutoGrid4 was used to create affinity grid maps for all the atom types. The affinity grids enclosed an area of 100 × 100 × 100 with 0.375 Å spacing, centered on the coordinates *x* = 86.3,  *y* = 51.2, and  *z* = 48.3. AutoDock4 (version 4.1) with the Lamarckian genetic algorithm was used with the following docking parameters: 100 docking runs, population size of 200, random starting position and conformation, translation step ranges of 2.0 Å, mutation rate of 0.02, crossover rate of 0.8, local search rate of 0.06, and 2.5 million energy evaluations [17]. The entire virtual screening experiment was performed on a cluster of 8 Intel Dual-Core 2.8 GHz computers using MOLA software [18]. Inhibition constants (*K*_*i*_) for all ligands were calculated by AutoDock4 as follows: *K*_*i*_ = exp⁡((Δ*G*∗1000)/(*R*cal∗TK)), where Δ*G* is the binding energy, *R*cal is 1.98719, and TK is 298.15. All figures with structure representations were prepared using PyMOL software [19].

## 3. Results and Discussion 

### 3.1. Synthesis

**Scheme 1:**
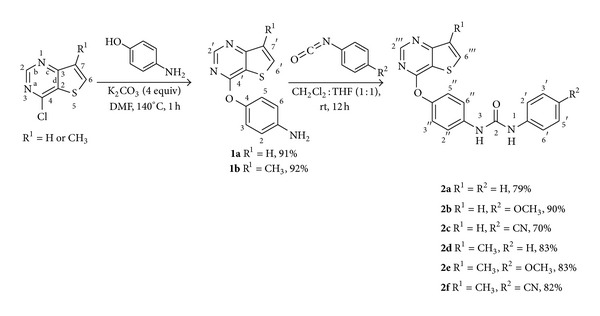
Synthesis of 1-aryl-3-[4-(thieno[3,2-*d*]pyrimidin-4-yloxy)phenyl]ureas **2a**–**2f**. The yields are presented as % of pure compounds.

We were able to promote the regioselective attack of the hydroxyl group of the 4-aminophenol in the chlorine nucleophilic displacement of two commercial 4-chlorinated thieno[3,2-*d*]pyrimidines, using stoichiometric amounts of the reagents, in the synthesis of the aminated compounds **1a** and **1b** in excellent yields (Scheme 1). This regioselectivity constitutes an important achievement that avoids the reaction of 4-nitrophenols followed by reduction of the nitro compounds to the corresponding amino compounds, as often described in the literature [12, 13]. With 4-aminophenol as a reagent, it is possible to use DMF as solvent and heat only at 140°C instead of diphenylether at 180°C which is needed for the nucleophilic displacement of the chlorine using 4-nitrophenol. The amino compounds **1a** and **1b**, obtained in one step, were then reacted with different arylisocyanates to give the corresponding new 1-aryl-3-[4-(thieno[3,2-d]pyrimidin-4-yloxy)phenyl]ureas **2a**–**2f** in high yields (Scheme 1). Compounds **1a**, **b**, and **2a**–**2f** were fully characterized by m.p.,  ^1^H,  ^13^C NMR, and mass spectrometry including HRMS.

### 3.2. Biological Evaluation

#### 3.2.1. Enzymatic Assays

The synthesized thieno[3,2-*d*]pyrimidine ureas **2a**–**f** were evaluated for their ability to interact with the VEGFR2 tyrosine kinase domain (Table 1), using an enzymatic FRET-based assay. Compounds **2a**, **2b**, and **2c** displayed the highest inhibition with IC_50_ values of 199, 188, and 150 nM, respectively. The presence of a methoxy group in the para position of the phenyl ring (**2b**) did not show a significant increase in VEGFR-2 inhibition activity relative to **2a**, while the presence of a nitrile group in the same position (**2c**) promoted a small increase in VEGFR-2 inhibition activity. These findings appear to suggest that the presence of small substituents in the *para* position of the phenyl group will not have a significant effect on VEGFR-2 potency of the compounds. When a methyl group is present in position 7 of the thieno[3,2-*d*]pyrimidine moiety (compounds **2d**–**f**), no VEGFR-2 inhibition activity was observed. This finding indicates that substitutions on the mentioned position are not favorable for this series of thieno[3,2-*d*]pyrimidine ureas, when considering VEGFR-2 inhibition activity.

#### 3.2.2. Cellular Assays

**Figure 2:**
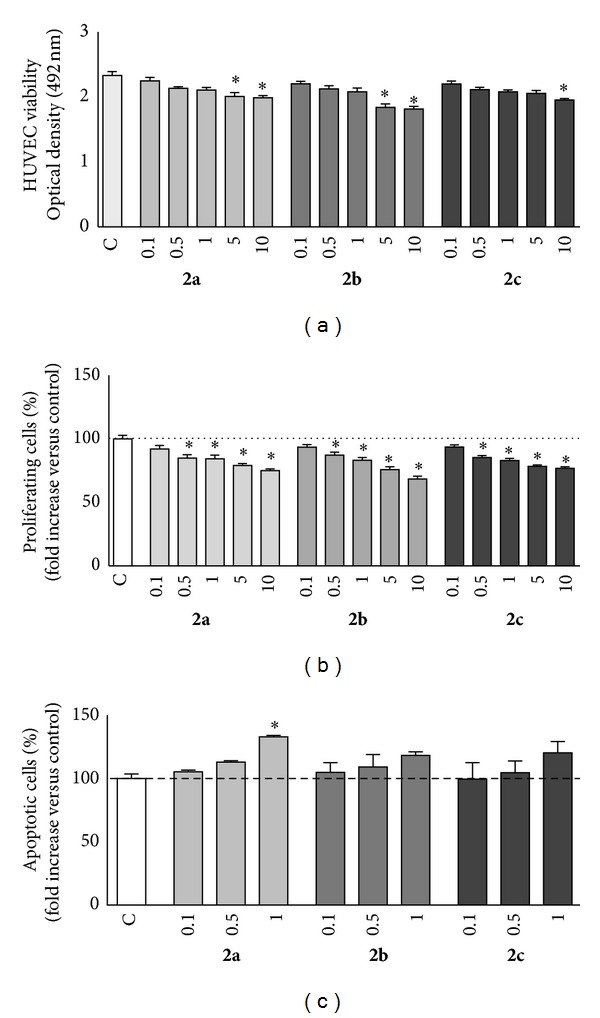
(a) Cell viability was evaluated in HUVECs upon treatment with **2a**, **2b**, and **2c** at 0.10, 0.50, 1.0, 5.0 and 10 *μ*M, or untreated (control, 0.1% DMSO) using MTS assay. (b) Cell proliferation was assessed by BrdU incorporation assay. Bars represent the percentage of proliferating cells when compared to control group, after incubation with anti-BrdU antibody by ELISA assay. (c) Apoptosis was assessed in HUVECs by TUNEL assay using **2a**, **2b**, and **2c** at 0.1, 0.5, and 1 *μ*M. Bars represent the percentage of apoptotic cells evaluated by the ratio between TUNEL-stained cells and DAPI-stained nuclei in every culture. Results are expressed as mean ± SEM of three independent experiments (5 < *n* < 10). *P < 0.05 versus control.

The best compounds in enzymatic assays (**2a**, **2b**, and **2c**) were studied at cellular level. Cell cytotoxicity was first analyzed by MTS assay in the HUVEC cultures upon treatment with 0.10–10 *μ*M of compounds **2a**–**c** (Figure 2(a)). Viability of HUVEC decreased at the highest concentration of **2c** (1.96 ± 0.02 O.D.) and at concentrations of 5.0 and 10 *μ*M for **2a** (2.00 ± 0.06 O.D. and 1.99 ± 0.03 O.D., resp.) and for **2b** (1.84 ± 0.05 O.D. and 1.82 ± 0.04 O.D., resp.) versus control (2.33 ± 0.06 O.D.). 

Then, the ability of compounds **2a**–**c** to inhibit VEGF-stimulated proliferation of HUVECs was evaluated using the BrdU incorporation assay (Figure 2(b)). Inhibition of the VEGFR-2 activity was strongly reflected at the cellular level, with all the three compounds showing a statistical significant inhibition activity against VEGF-stimulated HUVEC proliferation at 0.5 *μ*M (15.2 ± 2.7% for **2a**; 12.8 ± 2.4% for **2b**, and 14.7 ± 1.7% for **2c**) when comparing to control, which is consider to be 100.00 ± 2.8%.

The number of proliferating HUVECs decreased in a dose-dependent manner (Figure 2(b)).

Interestingly, incubation of HUVECs with compounds **2a**, **2b**, and **2c** for 24 h with concentrations 0.1, 0.5, and 1 *μ*M (due to cytotoxic effect for higher concentrations, Figure 2(a)) using the TUNEL assay resulted in an increase in apoptosis, reaching statistical significance only for **2a** at the highest concentration tested (32.8 ± 1.23% increase) comparing with untreated cells (Figure 2(c)).

**Figure 3:**
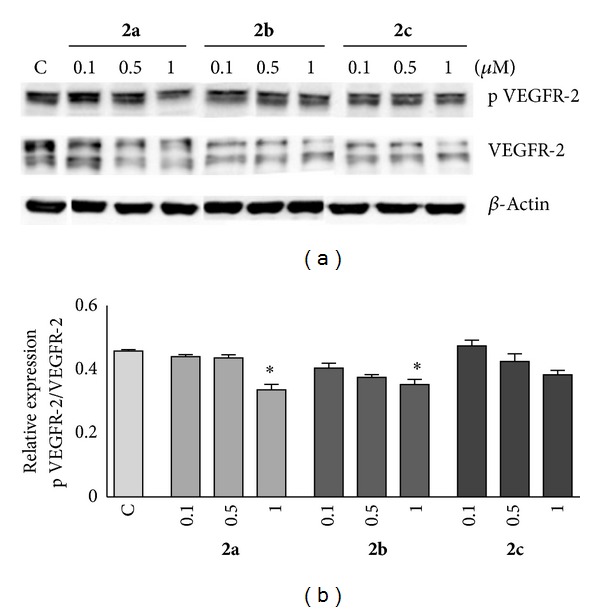
(a) Evaluation of phosphorylated and total VEGFR-2 expression in HUVECs after incubation with compounds **2a**, **2b**, and **2c** by western blotting. Representative bands obtained after immunostaining are shown. (b) Quantification of densitometry and mean relative intensity by comparison of the relative intensity of activated VEGFR-2 after normalization with total VEGFR-2 intensity. Data presented as mean ± SEM of two independent experiments. *P < 0.05 versus control.

To investigate the cellular inhibition of VEGFR-2 by the compounds in HUVEC cultures, immunoblotting assays for total and phosphorylated (active) VEGFR-2 were performed (Figure 3(a)). As illustrated in Figure 3(b), incubation with compounds **2a** and **2b** significantly decrease the formation of the active form of the receptor at 1 *μ*M concentration, when comparing with the expression of VEGFR-2 (*P < 0.05). Compound **2c** did not significantly inhibit the activation of the VEGFR-2, although a dose-dependent decrease was observed.

VEGF signaling pathway through the VEGFR-2 activation displays a crucial role in endothelial cells, namely, survival, proliferation, invasion, and apoptosis. Our data suggest that compounds **2a**–**c** exert direct actions in HUVECs by inhibiting their growth mainly at 0.5 and 1 *μ*M, slightly increasing the percentage of apoptotic cells without cytotoxic effects at the same range of concentrations. The referred cellular-based results are triggered by the inhibition of the phosphorylation (activation) of the VEGFR-2, which is overexpressed in several pathological conditions, such as cancer.

### 3.3. Molecular Modelling Studies

To better understand the molecular basis of compounds **2** observed VEGFR-2 inhibition activity, docking simulations using AutoDock4 [17] were carried out against the VEGFR-2 kinase domain (PDB code 2XIR). 

**Figure 4:**
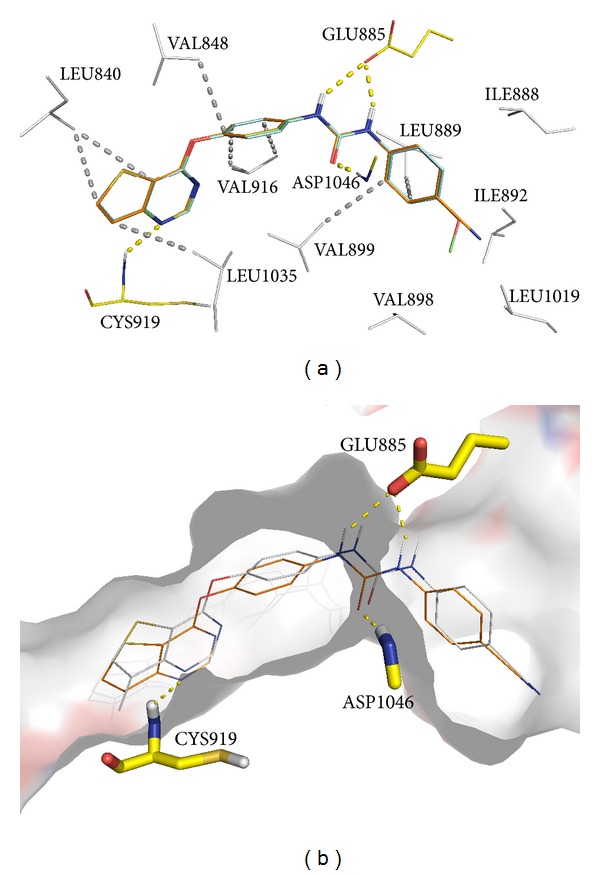
(a) Superimposition of the docking poses at the VEGFR-2 kinase domain for compounds: **2a** (cyan), **2b** (green) and **2c** (orange). (b) Surface representation of the VEGFR-2 kinase hinge with the docking poses of compounds **2c** (orange)and **2f** (light grey). H-bonds are depicted in dashed yellow lines (distances between 2.9 and 3.3 Å) and hydrophobic interactions are depicted in dashed white lines (distances between 3.5 and 4.0 Å). Important residues are drawn in sticks. Figures were prepared using PyMOL [19].

Figure 4(a) displays the superimposition of the best docked poses obtained for the most potent thieno[3,2-*d*]pyrimidine derivatives **2a**–**c**. They present similar docking poses, with the binding conformation stabilized mainly by the formation of a network of four H-bonds. The two N–H of the urea group form two H-bonds with the Glu885 carboxyl group, while the C=O bond of the urea group forms a third H-bond with the backbone N–H of Asp1046. These H-bonds are similar to the ones observed with other known VEGFR-2 inhibitors presenting a biphenyl urea moiety, including the drug Soranefib (Figure 1). A fourth H-bond is predicted between the N atom in position 4 of the thieno[3,2-*d*]pyrimidine moiety and the backbone N–H of Cys919. 

The terminal aryl group is positioned in a hydrophobic region formed by several hydrophobic residues (Ile888, Leu889, Ile892, Val898, Val 899, and Leu1019). From Figure 4(a), it is clearly observed that the 4-methoxyphenyl and 4-cyanophenyl substituents occupy part of a large hydrophobic pocket that is able to accommodate them, providing an explanation for compounds **2b** and **2c** being almost equipotent to **2a** that bears no substituents in the phenyl ring. Substitutions of hydrophobic groups in *ortho* or *meta* positions of the phenyl ring will probably be worth exploring for synthesis of even more potent compounds in this series.

The lack of inhibition by compounds **2d**–**f** for VEGFR-2 was analysed by superimposing the docked pose of compound **2c** (the most potent in the enzymatic assays, 150 nM) and the corresponding 7-methylated compound **2f** (Figure 4(b)). The methyl group seems to displace the thieno[3,2-*d*]pyrimidine ring away from Cys919. This displacement probably interferes with the formation of the H-bond between the N atom in position 4 of the thieno[3,2-*d*]pyrimidine ring and the backbone N–H of Cys919 (Figure 4(b)). These results demonstrate that this H-bond is an absolute requirement for VEGFR-2 inhibition potency of this thieno[3,2-*d*]pyrimidine series, and the methyl group in position 7 of compounds **2d**–**f** promotes loss of VEGFR-2 inhibition activity by impairing H-bond formation. We can also infer that other substitutions in position 7 of the thieno[3,2-*b*]pyridine are probably not desirable as they will also impair H-bond formation.

## 4. Conclusion 

In conclusion, we reported the synthesis of novel 1-aryl-3-[4-(thieno[3,2-*d*]pyrimidin-4-yloxy)phenyl]ureas **2a**–**2f** in high yields in two steps: the regioselective nucleophilic displacement of chlorine on two 4-chlorothieno[3,2-*d*]pyrimidines using 4-aminophenol and the reaction of the resultant 4-(thieno[3,2-*d*]pyrimidin-4-yloxy)anilines with arylisocyanates. Compounds **2a**–**c**, without the methyl group in position 7, were shown to be the most potent VEGFR-2 inhibitors presenting IC_50_ values between 150–199 nM in enzymatic assays. The cell culture assays enable us to identify the angiogenic steps targeted by the compounds **2a**–**c**. In fact, compounds were evaluated using a cell-based HUVEC assay and showed activity at the same concentration range (0.5–1 *μ*M) without affecting cell viability, inhibiting cell proliferation, and avoiding the formation of active VEGFR-2 specially in the case of compounds **2a** and **2b**, thus showing their anti-angiogenic potential. Compound **2a** increases also apoptosis at the highest concentration tested (1 *μ*M) using the TUNEL assay. By performing docking studies, the binding conformation and H-bond network of the most promising compounds **2a**–**c** were predicted. Based on these molecular modelling studies, several suggestions can be done for the synthesis of even more potent compounds.

## Figures and Tables

**Table 1:** Results of the VEGFR2 enzymatic inhibition assay using compounds **2a**–**f**.

Compounds	VEGFR-2 IC_50_ (*μ*M)^a^
**2a**	0.199
**2b**	0.188
**2c**	0.150
**2d**	>100
**2e**	>100
**2f**	>100

^a^Each IC_50_ determination is a result of four separate determinations.
